# Oligomer procyanidins (F2) repress HIF-1α expression in human U87 glioma cells by inhibiting the EGFR/ AKT/mTOR and MAPK/ERK1/2 signaling pathways *in vitro* and *in vivo*

**DOI:** 10.18632/oncotarget.19654

**Published:** 2017-07-28

**Authors:** Hong-Li Zheng, Li-Hui Wang, Bao-Shan Sun, Yi Li, Jing-Yu Yang, Chun-Fu Wu

**Affiliations:** ^1^ Department of Pharmacology, Shenyang Pharmaceutical University, Shenyang, P.R. China; ^2^ Department of Enology, Shenyang Pharmaceutical University, Shenyang, P.R. China; ^3^ Benxi Institute of Pharmaceutical Research, Shenyang Pharmaceutical University, Benxi, P.R. China; ^4^ Viticultural and Enological Research Unit-National Institute for Agricultural and Veterinary Research, Dois Portos, Portugal

**Keywords:** F2, HIF-1α, angiogenesis, invasion, U87 glioma cell

## Abstract

Hypoxia-inducible factor-1 (HIF-1) is over-expressed in gliomas and has become one of the most compelling tumor targets. In this study, we found that oligomer procyanidins (F2) can suppress the expressions of HIF-1α and its target genes in U87 cells, and also down-regulate the EGFR/PI3K/AKT/mTOR and MAPK/ERK1/2 pathways *in vitro* and *in vivo*. Furthermore, hypoxia-induced formation of tubular structures by human umbilical vascular endothelial cells and the migration and invasion of U87 cells could be inhibited by F2 in a HIF-1 dependent manner. Moreover, in a U87 xenograft tumor model, F2 significantly reduced intra-tumor vessel density and cell proliferation and finally retarded tumor growth, indicating that F2 may be a potential HIF-1 inhibitor and serve as one of candidates for glioma therapy.

## INTRODUCTION

Glioma is the most aggressive form of primary brain tumor with high mortality and morbidity [1]. A predominant feature of glioma is the hypoxic tumor microenvironment [2], which is a powerful driving force for malignant progression by activating adaptive transcriptional programs-hypoxia-inducible factor-1 (HIF-1) [3]. HIF-1 is a heterodimeric transcription factor consisting of a hypoxia-inducible α subunit (HIF-1α) and a constitutively expressed β subunit (HIF-1β), and regulates pleiotropic effects including promoting invasion and angiogenesis, the switch to glycolytic metabolism, and up-regulation of cell survival-related molecules [4]. As the only oxygen-regulated subunit, HIF-1α activation is also controlled by multiple oncogenic pathways, which involve loss of tumor suppressor genes, such as PTEN and growth factor signaling [5], both of which are common alterations found in gliomas [6].

Significant up-regulation of HIF-1α expression has been demonstrated in glioma cells and clinical specimens [7, 8], and there is accumulating evidence that down-regulation of HIF-1α can inhibit tumor angiogenesis and cell invasion [2, 9]. Suppression of HIF-1α has been shown to reduce VEGF (vascular endothelial growth factor) expression and angiogenesis in U251 cells [10]. In addition, knock down of HIF-1α in glioma cells impairs their ability to migrate and invade [11], and also increases the sensitivity of cells to chemotherapy and inhibits the self-renewal of glioma stem cells (GSCs) [12]. Hence, HIF-1α is an attractive target for the development of novel anti-glioma agents.

Oligomer procyanidins (F2, degree of polymerization =2-15) isolated from grape seeds (Supplementary Figure 1A) have been found to have multiple effects including anti-inflammatory, anti-oxidant and anti-tumor properties [13]. Previously, we reported that the most sensitive tumor cell line of F2 was U87 cell line, and it could inhibit U87 cell proliferation by inducing a non-apoptotic cell death phenotype resembling paraptosis [14]. At a non-cytotoxic concentration, F2 selectively suppressed the function of FPR1 (formyl peptide receptor 1) and the FPR1-mediated signaling pathway in a manner of a partial agonist in U87 cells [15], and it also inhibited the HIF-1 pathway in U251 glioma cells [16]. Hence, to further understand and identify its underlying anti-glioma effect and mechanism, we investigated the influence of F2 on HIF-1 pathway and it mediated biological effects in U87 glioma cells.

## RESULTS

### F2 inhibits HIF-1α expression in the U87 glioma cell line

**Figure 1:**
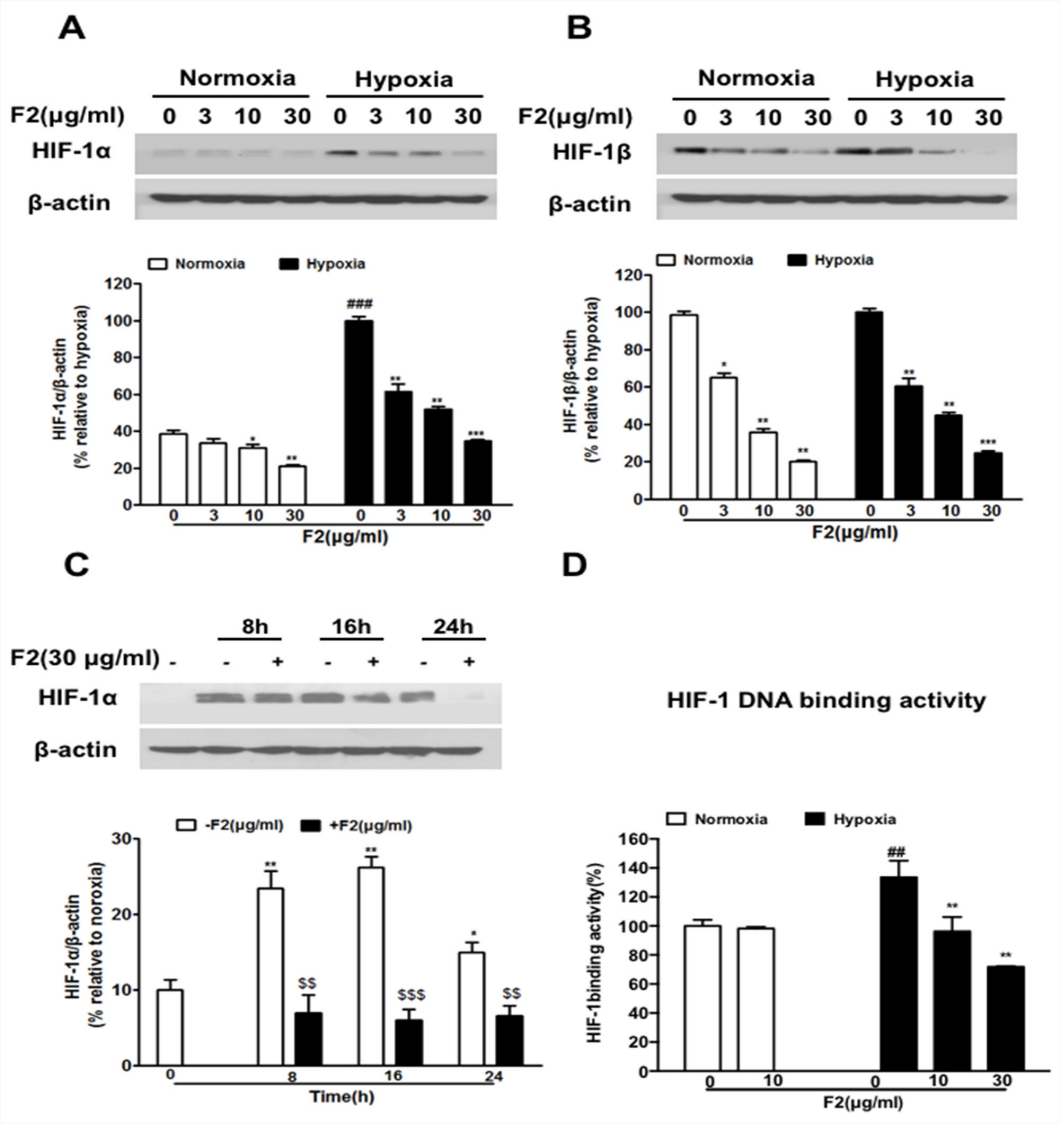
The effects of F2 on HIF-1α and HIF-1β expression in U87 cells **(A, B)** Western blot analyses of HIF-1α and HIF-1β were done using total proteins extracted from U87 cells incubated under normoxic or hypoxic conditions for 16 h in the presence or absence of the indicated concentrations of F2. Values are means ± SEM of three experiments. ### P<0.0001 compared with the normoxic group; * P<0.05, **P<0.01 and ***P<0.001 compared with the group untreated with F2. **(C)** U87 cells were treated with F2 (30 μg/ml) for 8 h, 16 h and 24 h under normoxic and hypoxic conditions, and total proteins were collected for western blot analysis. Values are means ± SEM of three experiments. * P<0.05, **P<0.01 compared with normoxic group. $$P<0.01 and $$$P<0.001 compared with the group untreated with F2. **(D)** HIF-1α DNA binding activity assay was done using nuclear proteins extracted from U87 cells incubated under normoxic or hypoxic conditions for 16 h in the presence or absence of the indicated concentrations of F2. Values are means ± SEM of three experiments. ## P<0.01 compared with the normoxic group; ** P<0.01 compared with the hyooxic group on time point 0.

Firstly, we examined the effect of F2 on the expression of HIF-1α and HIF-1β in U87 cells. As shown in Figure 1A and 1B, F2 significantly inhibited the expression of HIF-1α and HIF-1β protein (P<0.001, P<0.05) in a concentration-dependent manner (3-30 μg/ml) without obvious cytotoxic effects (Supplementary Figure 1B) (P>0.05), and it also decreased the level of HIF-1α protein at all time points we tested (Figure 1C) (P<0.001). We further found that F2 significantly decreased the HIF-1 DNA binding activity(Figure 1D) (P<0.01). Since HIF-1α is the only oxygen regulatory subunit of HIF-1 and participates in the development and progression of glioma, we focused our subsequent research on the HIF-1α subunit.

### F2 does not influence the stability, degradation or transcription of HIF-1α

**Figure 2:**
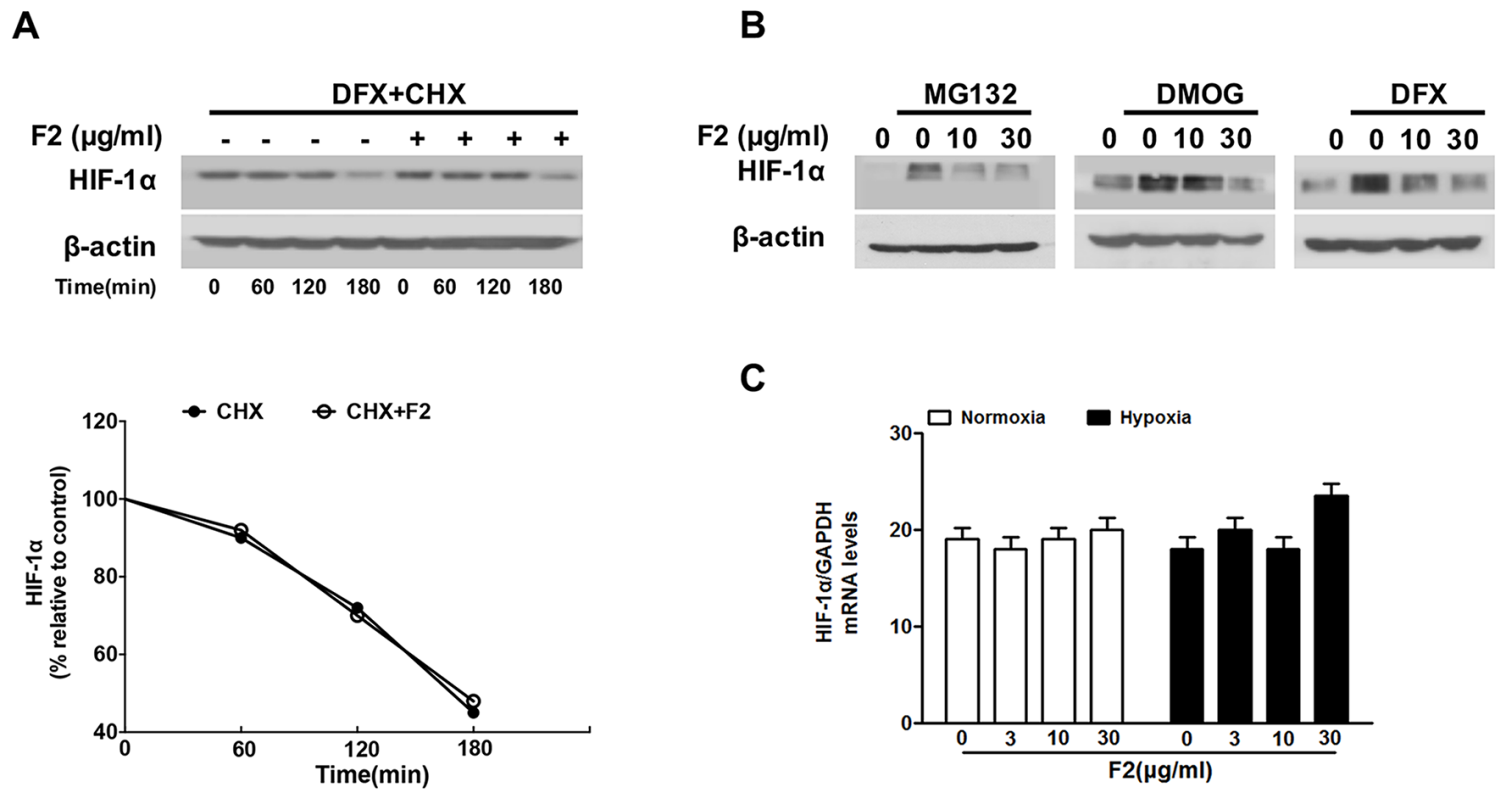
The effects of F2 on HIF-1α half-life, degradation and transcription **(A)** Cells were treated with DFX (100 μM) in the presence or absence of F2 (10 μg/ml) for 4 h, and then CHX (40 μg/ml) was added for the indicated time. Values were normalized to β-actin and expressed as a percentage relative to time 0, which was considered equal to 100 %. **(B)** U87 cells were treated with MG132 (20 μM), DMOG (100 μM) or DFX (100 μM) for 8 h under normoxic conditions in the presence or absence of the indicated concentrations of F2, then total proteins were collected for western blotting. **(C)** Real-time PCR analysis of HIF-1α mRNA using total RNA extracted from U87 cells incubated under hypoxia for 16 h in the presence or absence of the indicated concentrations of F2.

To further investigate how F2 decreases the HIF-1α level in U87 cells, we firstly tested whether the degradation rate (half-life) of HIF-1α protein was affected by F2 in the presence of CHX, a protein translation inhibitor. U87 cells were pre-incubated with DFX (100 μM) for 4 h to induce steady-state levels of HIF-1α, then treated with 40 μM of CHX with or without 10 μg/ml of F2. Results showed that the half-life of HIF-1α was unchanged before and after treatment with F2 (Figure 2A).

Next, we examined whether F2 regulated HIF-1α protein degradation via the hydroxylation-proteasome pathway, which is the main HIF-1α protein degradation pathway [17]. To address this question, we treated U87 cells with a proteasome inhibitor (MG132) or hydroxylase inhibitors (DMOG, DFX) to test whether they interrupted HIF-1α degradation. As shown in Figure 2B, F2 suppressed the accumulation of HIF-1α protein despite the inhibition of hydroxylase or proteasome activity, indicating that it decreases the expression of HIF-1α protein through a pathway independent of hydroxylation-proteasome-mediated degradation. Real-time PCR experiments showed that the levels of HIF-1α mRNA were not affected by F2 under normoxic or hypoxic conditions (Figure 2C). Overall, these results suggest that F2 probably decreases the expression of HIF-1α protein at the translational level.

### F2 suppresses HIF-1α translation through the EGFR/PI3K/AKT/mTOR and MAPK/ERK1/2 pathways

**Figure 3:**
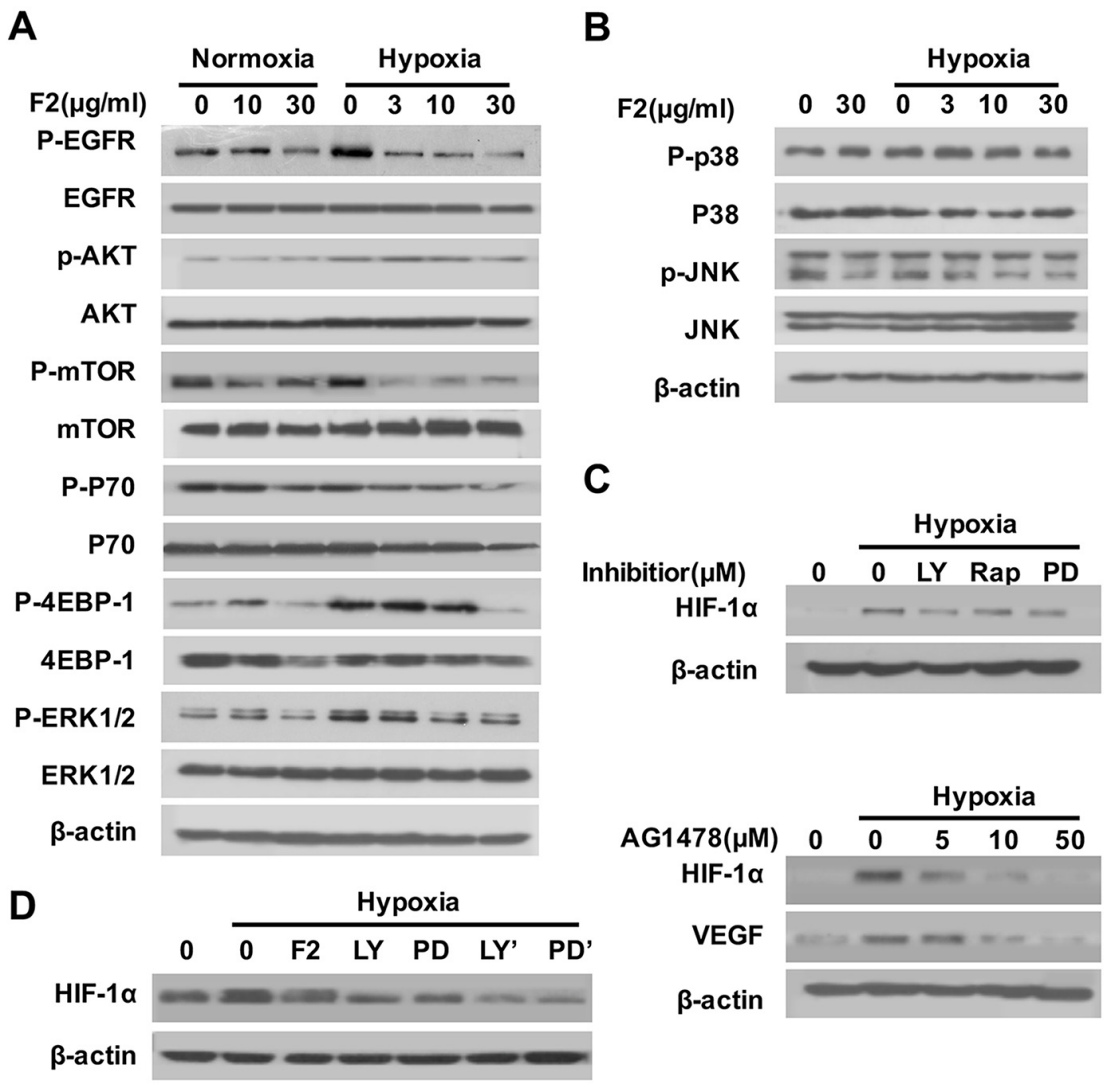
The effects of F2 on the EGFR/PI3K/AKT/mTOR and MAPK pathways **(A, B)** Effects of F2 on hypoxia-induced signaling pathways related to the expression of HIF-1α in U87 cells. Cells were pretreated with the indicated concentrations of F2 for 1 h, then incubated under normoxic and hypoxic conditions for 1 h. Total proteins were then collected for western blotting using antibodies as indicated. **(C)** Effects of inhibitors on hypoxia-induced expression of HIF-1α in U87 cells. Cells were pretreated with LY (LY294002, 100 μM), Rap (Rapamycin, 100 nM), PD (PD98059, 100 μM) and AG1478 for 1 h, and then incubated under normoxic or hypoxic conditions for 16 h. Total proteins were subjected to western blotting using antibodies against HIF-1α. **(D)** Cells were pretreated with or without LY (LY294002, 100 μM) or PD (PD98059, 100 μM), then co-treated with or without F2 (10 μg/ml) for 16 h under hypoxic conditions. LY’ and PD’ indicate cells treated with inhibitors and F2. Total proteins were collected for western blot analysis.

The EGFR/PI3K/AKT/mTOR and MAPK pathways are the key upstream pathway that regulate the HIF-1α translation and synthesis [18]. To explore whether the inhibition of HIF-1α protein by F2 is linked to the suppression of either one or both pathways, we examined the effect of F2 on the levels of the total and phosphorylated forms of EGFR, AKT, mTOR (P70 and 4EBP-1), ERK1/2, p38 and JNK. Results showed that hypoxia greatly increased the levels of phosphorylated EGFR, AKT, mTOR, P70, 4EBP-1 and ERK1/2, but not of phosphorylated p38 and JNK (Figure 3A and 3B), indicating that the EGFR/PI3K/AKT/mTOR and ERK1/2 pathways participate in hypoxia-induced HIF-1α expression in U87 cells. And F2 (3-30 μg/ml) could inhibit the phosphorylation of EGFR, AKT, mTOR, P70, 4EBP-1 and ERK1/2 in a concentration-dependent manner (Figure 3A), but failed to influence the phosphorylation of p38 and JNK (Figure 3B). To further confirm whether the EGFR/AKT/mTOR and ERK1/2 pathways are required for the hypoxia-induced HIF-1α expression in U87 cells, we exposed cells to various kinase inhibitors. The specific inhibitors of EGFR (AG1478), PI3K (LY294002), mTOR (Rapamycin) and MEK1 (PD98059) decreased the hypoxia-induced expression of HIF-1α and VEGF proteins in U87 cells (Figure 3C). Moreover, pretreatment of U87 cells with LY294002 or PD98059 for 1 h before adding F2 (10 μg/ml) caused a stronger reduction in HIF-1α expression than F2 or inhibitor alone (Figure 3D), suggesting that both the AKT and ERK1/2 pathways are involved in the inhibition of HIF-1α expression by F2. It is well known that phosphorylation of 4EBP-1 and P70 are important steps for the initiation of HIF-1α protein translation [17]. In our study, the phosphorylation of both 4EBP-1 and P70 were inhibited by F2 in U87 cells through down-regulation of the AKT and ERK1/2 pathways (Figure 3A), further confirming that F2 inhibits HIF-1α protein expression in U87 cells, probably by influencing the protein translation through the EGFR/PI3K/AKT/mTOR and MAPK/ERK1/2 pathways.

### F2 decreases HIF-1α expression induced by EGF and FBS

**Figure 4:**
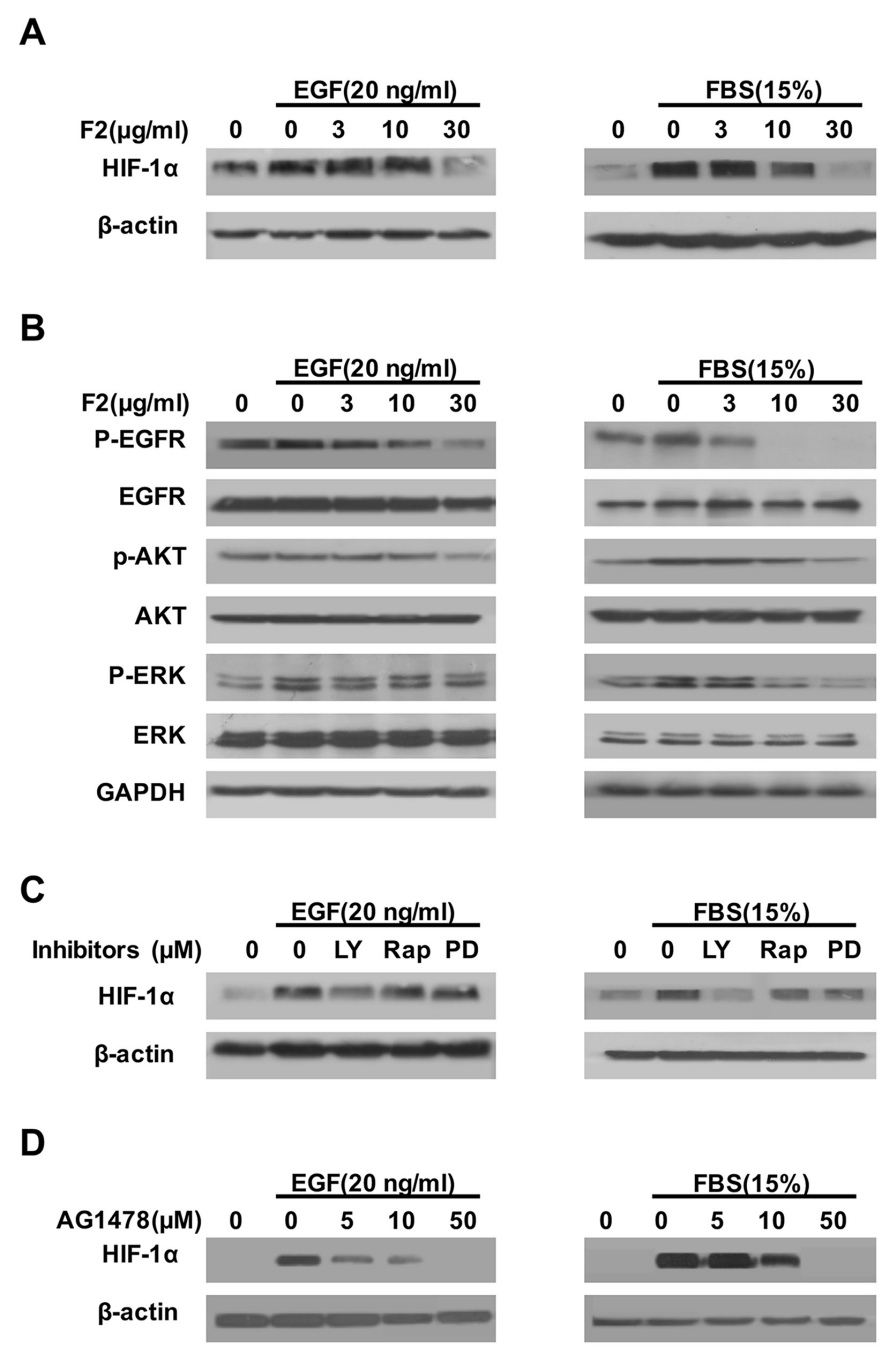
The effects of F2 on EGF- and FBS-induced HIF-1α expression **(A, B)** Effects of F2 on EGF- or FBS-induced signaling related to the expression of HIF-1α in U87 cells. Cells starved for 24 h were pretreated with the indicated concentrations of F2 for 1 h, followed by incubation with EGF or FBS for16 h or 1 h respectively. HIF-1α and the phosphorylated and total levels of EGFR, ERK, and AKT were determined by western blotting. **(C)** Effects of kinase inhibitors on EGF- or FBS-induced expression of HIF-1α in U87 cells. Cells starved for 24 h were pretreated with LY (LY294002, 100 μM), Rap (Rapamycin, 100 nM), PD (PD98059, 100 μM) and AG1478(5-50 μM) for 1 h, then incubated with EGF or FBS for16 h. Total protein extracts were subjected to western blotting using antibodies against HIF-1α or β-actin. **(D)** U87 cells were pre-treated with the indicated concentrations of AG1478 for 1 h, then incubated with EGF or FBS for 16 h. Total protein extracts were analyzed by western blotting with the indicated antibodies.

Previous studies showed that activation of the EGFR/PI3K/AKT and MAPK/ERK1/2 pathways by growth factors (EGF, IGF) and serum could up-regulate the HIF-1α protein [19, 20]. In this study, F2 (3-30 μg/ml) also inhibited the expression of HIF-1α protein in U87 cells induced by FBS (15%) or EGF (20 ng/ml) in a concentration-dependent manner (Figure 4A). Moreover, pretreatment of U87 cells with F2 decreased the levels of phosphorylated EGFR, AKT and ERK1/2, which paralleled the inhibitory effect of F2 on HIF-1α protein expression (Figure 4B). To further confirm our findings, we pretreated U87 cells with LY294002, Rapamycin, PD98059 or AG1478, then exposed the cells to FBS or EGF for 16 h before testing the level of HIF-1α. The results showed that the accumulation of HIF-1α protein induced by FBS and EGF was greatly inhibited by LY294002, Rapamycin, PD98059 and AG1478 (Figure 4C and 4D) respectively, further suggesting that EGFR/PI3K/AKT and ERK1/2 pathways are really the major regulator of HIF-1α expression in U87 cells.

### F2 inhibits hypoxia-induced VEGF expression and angiogenesis *in vitro*

**Figure 5:**
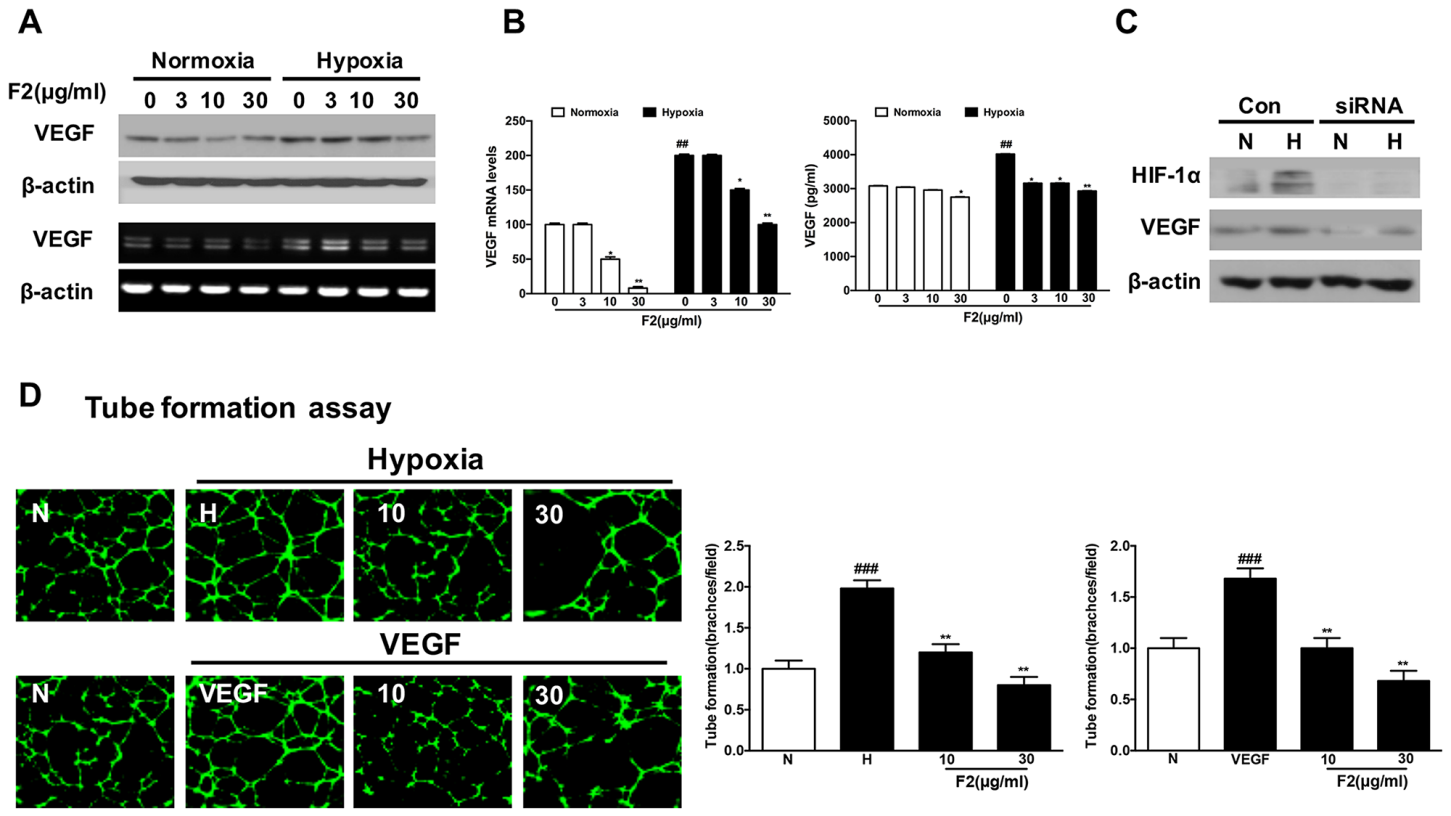
The effects of F2 on VEGF expression and formation of tubular structures by endothelial cells **(A, B)** VEGF protein expression was evaluated by western blot analysis of protein extracts from U87 cells (A, upper panel) and ELISA in the culture supernatant (B, right panel) after incubation under hypoxia in the presence or absence of the indicated concentrations of F2. RT-PCR (A, lower panel) and real-time PCR analysis (B, left panel) of VEGF mRNA were carried out using total RNA prepared from U87 cells incubated under hypoxia for 16 h in the presence or absence of the indicated concentrations of F2. **(C)** Western blot analysis of HIF-1α and VEGF was carried out using total proteins from U87 cells transfected with HIF-1α siRNA or control siRNA and treated under normoxic and hypoxic conditions for 16 h. **(D)** The tube formation assay was performed as described in the Methods section. Values are means ± SEM of three experiments. ### P<0.01 compared with the normoxic group; ** P<0.01 compared with the hypoxic group.

As an important regulator of angiogenesis, VEGF is mainly controlled at the transcriptional level by HIF-1 [21]. Here, the levels of VEGF mRNA and protein were significantly inhibited by F2 under normoxic and hypoxic conditions (Figure 5A and 5B). And F2 also dramatically suppressed HUVEC tube formation induced by hypoxia and VEGF (10 ng/ml) (Figure 5D) (P<0.01), which was further confirmed by the results of real-time cell analysis (RTCA) and wound-healing assays (Supplementary Figures 1C and 2A). Moreover, knockdown of HIF-1α decreased VEGF expression in U87 cells under normoxic and hypoxic conditions (Figure 5C), indicating that VEGF is regulated by HIF-1α, and inhibition of angiogenesis is one anti-glioma mechanism of F2 in U87 cells.

### F2 inhibits hypoxia-induced MMP-2 expression and U87 cell migration and invasion

**Figure 6:**
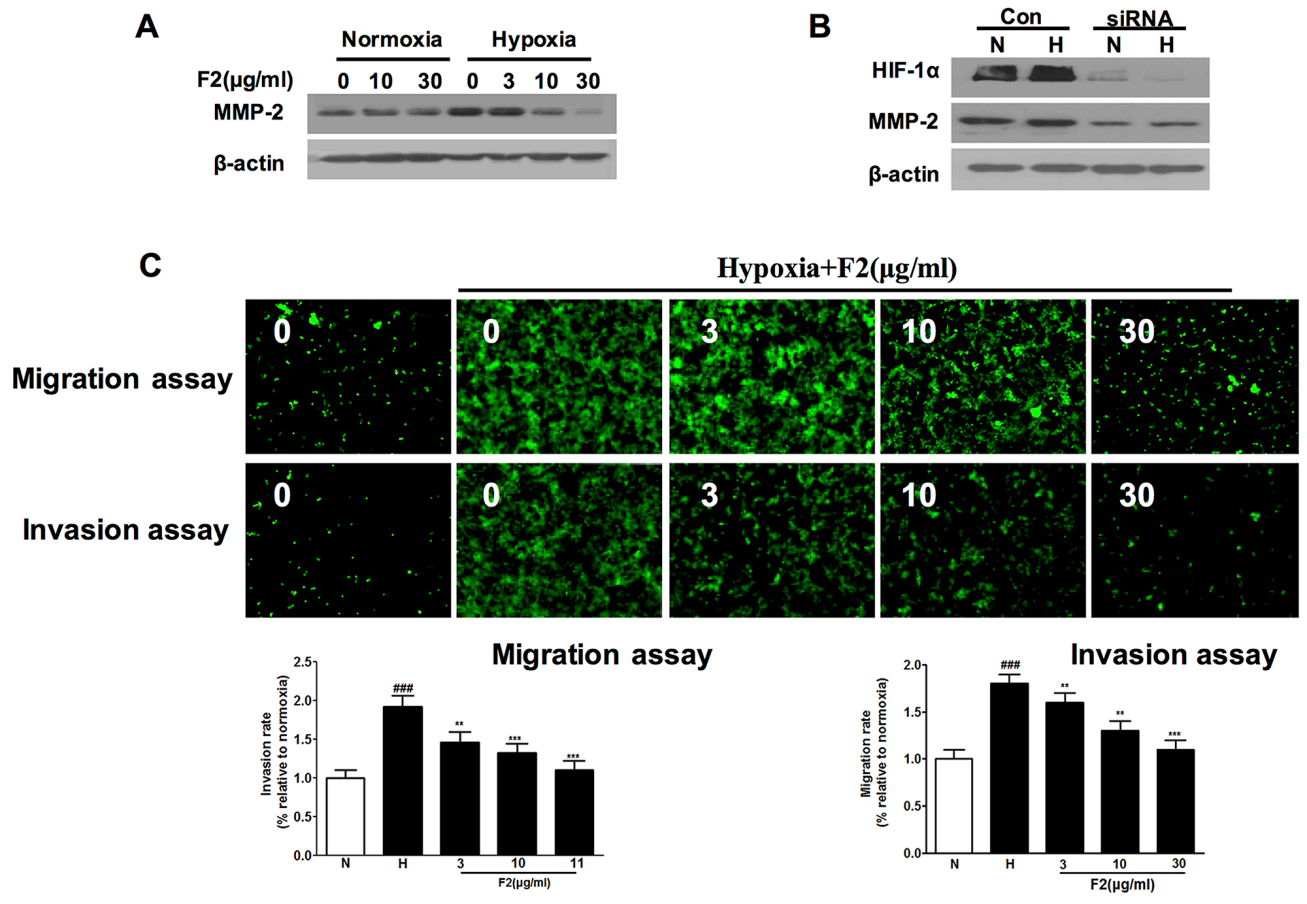
The effects of F2 on expression of MMP-2 and migration and invasion of U87 cells **(A)** Western blot analysis of MMP-2 levels in extracts of U87 cells incubated under normoxic or hypoxic conditions with the indicated concentrations of F2. **(B)** Western blot analysis of MMP-2 levels in cells transfected with HIF-1α or control siRNA incubated under hypoxia for 16 h. **(C)** Migration and invasion assays were done as described in the Methods section. Values are means ± SEM of three experiments. ### P<0.001 compared with normoxic group; ** P<0.01 and ***P<0.001 compared with hypoxic group.

In addition to angiogenesis, hypoxia also stimulates the cancer cell migration, invasion and metastasis [8, 11]. It has been reported that hypoxia induced glioma cell migration by increasing the activity and protein level of MMP-2 [22]. In our study, we found that F2 not only decreased the expression of MMP-2 under normoxic and hypoxic conditions (Figure 6A), but also significantly inhibited hypoxia-induced U87 cell migration and invasion (Figure 6C, Supplementary Figure 2B) (P<0.01). Moreover, knockdown of HIF-1α protein decreased the level of MMP-2 protein in U87 cells (Figure 6B), which is highly consistent with the previously reported result [11]. And these findings suggest that inhibition of cell migration and invasion may be another mechanism by which F2 has an anti-glioma effect.

### F2 retards U87 tumor growth, probably by inhibiting the HIF-1 pathway *in vivo*

**Figure 7:**
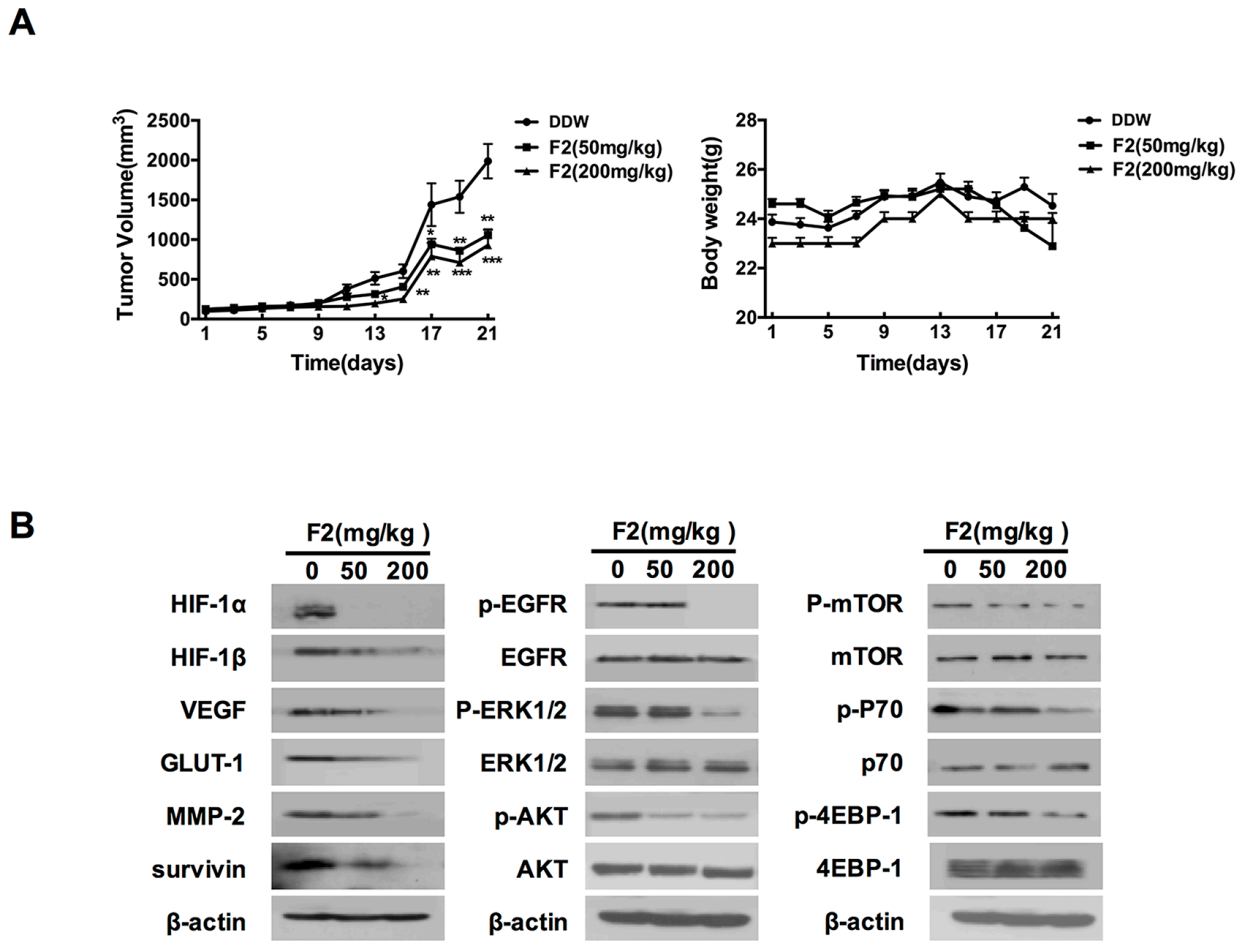
The effects of F2 on tumor growth and the HIF-1 pathway *in vivo* **(A)** Relative tumor volume and body weight in U87 xenograft mice treated with F2 (mg/kg i.g.). Values are means ± SE of three experiments. ** P<0.01 and **P<0.001 compared with control group. **(B)** Western blot analysis of HIF-1α, proteins encoded by HIF-1 target genes and proteins related to the EGFR signaling pathway in tumors from U87 xenograft mice treated with or without the indicated concentrations of F2 (mg/kg i.g.).

The *in vivo* efficacy of F2 against gliomas was investigated in U87 cells xenograft tumors in nude mice. As shown in Figure 7A, oral gavage feeding of F2 (50, 200 mg/kg) significantly inhibited the growth of U87 xenograft tumors in a dose-dependent manner (P<0.05), and did not cause any observable toxic effects or body weight decline (P>0.05) based on the observations of body weight and dietary consumption.

**Figure 8:**
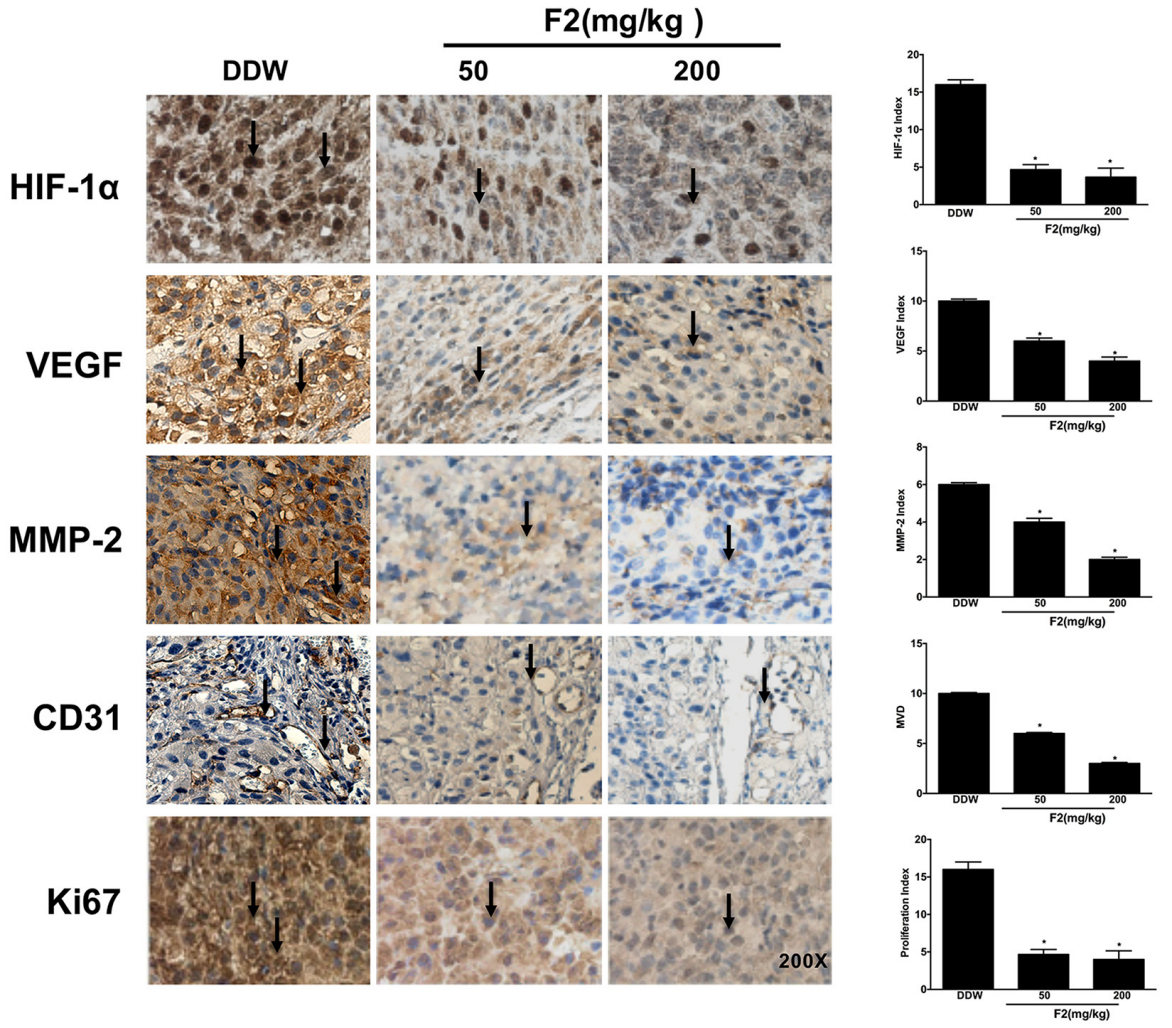
Effects of F2 on tumor-related biomarkers *in vivo* Immunohistochemical analysis of tumor-related proteins (HIF-1α, VEGF, MMP-2, CD31 and KI67) in tumor sections from U87 xenograft mice treated with F2 (mg/kg i.g.). Red arrows indicate the related protein detected in the tissue samples. Values are means ± SEM of three experiments. * P<0.05 compared with DDW group.

To determine whether the changes that F2 affected the expression of HIF-1α and its target genes *in vitro* also occur *in vivo*, we examined the protein levels in U87 xenograft tissue samples from animals by western blot and immunohistochemical analysis. As shown in Figures 7B and 8, oral gavage feeding of F2 at doses of 50 and 200 mg/kg significantly decreased the levels of HIF-1α (P<0.05), HIF-1β, VEGF (P<0.05), MMP-2 (P<0.05) and other HIF-1 target genes (GLUT-1, survivin). Consistent with the *in vitro* findings, the EGFR/PI3K/AKT/mTOR and MAPK/ERK1/2 pathways were also inhibited by F2 *in vivo* (Figure 7B). We also found that the expression of HIF-1α occurred mainly in the nuclei in the F2-treated tumors, suggesting that F2 has little effect on the sub-cellular localization of HIF-1α. Overall, these findings confirm that F2 may target HIF-1α and induce anti-glioma effects in both cell culture and tumor xenograft models.

To further assess the *in vivo* anti-proliferative and anti-angiogenic effects of F2, tumor samples were analyzed by immunohistochemistry for Ki67 (a cell proliferation biomarker) and CD31 (a marker of endothelial cells). As expected, the levels of Ki67 and CD31 were decreased in the F2-treated tumors (Figure 8) (P<0.05). In addition, F2 also decreased the level of Bcl-2 *in vitro* and *in vivo* (data not shown), which is also a target gene of HIF-1. Taken together, *in vitro* and *in vivo* results show that F2 can effectively inhibit U87 glioma cell proliferation, angiogenesis, migration and invasion probably via the HIF-1 pathway *in vitro* and *in vivo*.

## DISCUSSION

HIF-1α is highly expressed in gliomas and closely related to tumor progression, invasion and abnormal vascular development [3] and has been an attractive target for the glioma diagnosis and therapy [9]. In this study, we demonstrate that oligomer procyanidins (F2) isolated from grape seeds could decrease the expression of HIF-1α in U87 cells and inhibit its related angiogenesis and cell invasion. In the xenograft tumors model, F2 also decreased Ki67 and CD31 in tumor tissue, and significantly inhibited tumor growth and decreased the vessel density.

It is well known that multiple genes expression involved in tumor angiogenesis and cell invasion were HIF-1 targets [23, 24]. Here, F2 also inhibited the expression of VEGF and MMP-2 in a HIF-1α dependent manner, and finally inhibited the hypoxia-induced tube formation and invasion of HUVECs, and migration and invasion of U87 cells. In addition, iNOS (inducible nitric oxide synthase), a regulator of hypoxia-induced VEGF expression [25], was also inhibited by F2 in U87 cells (data not shown). These data suggest that the inhibition of F2 on HIF-1 mediated angiogenesis and cell invasion may be the major anti-glioma effects.

As the major upstream regulator of HIF-1α expression, EGFR/PI3K/AKT/mTOR and MAPK/ERK1/2 pathways were simultaneously suppressed by F2 in U87 cells and xenograft tumor model. Interestingly, previously results showed that F2 was a partial agonist of FPR1, and could inhibit the function of FPR1 and cell chemotaxis and invasion in U87 cells [15]. And FPR1 could trans-activate the EGFR and induce the HIF-1α and VEGF expression, and mediate the angiogenesis [26]. Thence, EGFR signaling pathway is certainly the major and probably the unique pathway for the anti-glioma effect of F2. However, much more detail effects of F2 on EGFR function, such as its tyrosine kinase activity, and the FPR1-EGFR-HIF-1 loop are forwardly needed to explore, and also the HIF-1 independent pathways (NF-κB, STAT3), they are all the downstream signaling of EGFR pathway.

In summary, F2 could inhibit the HIF-1 signaling pathway in U87 cells *in vitro* and *in vivo*. And combination with our previous studies, we can conclude that F2 could display multiple anti-glioma effects involved in cell survival, death, angiogenesis and invasion, indicating that F2 might be one of the promising therapeutic or assistant agents for the treatment of glioma in the future.

## MATERIALS AND METHODS

### Reagents and cell lines

#### Reagents

F2 was prepared from grape seeds as described previously [27]. Briefly, F2 was extracted from grape seed methanolic extract and evaporated at less than 30 °C to dryness, then dissolved in sterile water prior to lyophilization [28]. The purity of F2 was 93.0 ± 1.3%. F2 was stored at -20 °C until used and was dissolved in double distilled water (DDW) for all the experiments.

Rapamycin, LY294002, cycloheximide (CHX), MG132, AG1478 and DMOG were purchased from Sigma (St. Louis, MO). HIF-1α siRNA and control siRNA were purchased from Santa Cruz Biotech (CA, USA).

#### Cell lines

U87 human glioma cells (American Type Culture Collection, ATCC) were grown in DMEM (high glucose) with 10% heat-inactivated FBS (Gibco BRL, Grand Island, NY), 100 μg/ml streptomycin and 100 IU/ml penicillin in a humidified incubator containing 21% O_2_ and 5% CO_2_ in air (referred to as normoxic conditions). For cells cultured under hypoxia, cells were grown in a chamber containing 1% O_2_, 5% CO_2_ and 94% N_2_ at 37 °C. Human umbilical vascular endothelial cells (HUVECs) (National Center for Medical Culture Collection) were grown in RPMI 1640 medium containing 10% heat-inactivated fetal bovine serum, 100 μg/ml streptomycin and 100 IU/ml penicillin. All cell lines used were between passages 3 and 8 for each experiment and were demonstrated to be free of mycoplasma using Mycoplasma PCR Detection Kit (Sigma, USA).

### Western blot analysis

Western blotting was performed as described previously [15]. In brief, equal amounts of total protein extracts were fractionated by 4-15% SDS-PAGE and electrically transferred onto polyvinylidene difluoride (PVDF) membranes. Mouse or rabbit primary antibodies and appropriate horseradish peroxidase (HRP)-conjugated secondary antibodies were used to detect the designated proteins. Membrane-bound secondary antibodies were detected with ECL reagents and exposed to X-ray films. Results were normalized to the internal control (β-actin).

### Quantitative PCR analysis

Total RNAs were isolated using an RNeasy Mini Kit (Qiagen, Inc.) following the manufacturer’s instructions. RNAs were reverse transcribed with a RevertAid First Strand cDNA Synthesis Kit (TOYOBO, Japan) [14]. For quantitative PCR, analysis was carried out using iQ SYBR Green Supermix and a CFX96 Real-Time PCR Detection System (Bio-Rad) as instructed by the manufacturer. GAPDH was used as an internal control.

### Transwell cell invasion and migration assay

Cell invasion and migration assays were performed using a modified Boyden chamber. Cells (10-20×10^4^) were plated in RPMI 1640 medium in the upper chamber of the Transwell (8 μm pore, Corning Costar, Corning, NY) pre-coated with or without Matrigel (Becton-Dickinson, Bedford, MA). Serum free 1640 RPMI medium with or without of F2 (3-30 μg/ml) was added to the upper and lower Transwell chambers. After 12 h or 16 h, non-migrated cells were removed with a cotton swab and migrated cells were stained and examined using a high-content instrument (MD, USA).

### Tube formation assay

50 μl Matrigel™ (BD, Bioscience, Bedford, MA) mixed with serum-free 1640 RPMI medium (1:1) were placed into each well of a 96 well-plate and allowed to polymerize by incubation at 37 °C for 30 min. 100 μl of HUVECs (5×10^4^/well) were seeded on the gel with or without F2 (3-30 μg/ml), then incubated at 37 °C for 12 h. Cells were stained and examined using a High Content Screening System (MD), and tube formation (branches/field) was examined and quantified.

### Human glioma xenograft mouse model

To determine the *in vivo* anti-tumor activity of F2, viable U87 cells (5×10^6^/100 μl PBS per mouse) were mixed with 50% Matrigel, and then subcutaneously (s.c.) injected into the right flank of 7-8 week old male SCID mice. When the average s.c. tumor volume reached 100 mm^3^, the mice were randomly divided into three treatment groups (n=6-8), including control (DDW, i.g.), F2 (50 mg/kg/day, i.g.) and F2 (200 mg/kg/day, i.g.) for 5 days/wk. Tumor size and body weight were measured once every two days. After 21 days, the mice were sacrificed, and the tumors were excised and stored at -80 °C until further analysis. This study was performed in strict accordance with the recommendations in the Guide for the Care and Use of Laboratory Animals of the National Institutes of Health. The protocol was approved by the Committee on the Ethics of Animal Experiments of the Shenyang Pharmaceutical University.

### Immunohistochemical analysis

Representative tumor tissues (four from each group) were harvested and fixed in 10% neutral buffered formalin at 4 °C for 12 h. All tissues were paraffin embedded. Sections (5 μm) were micro-waved in citrate buffer and were stained with antibody against HIF-1α (Novus biological, NB100-105), Matrix metalloproteinase-2 (MMP-2) (Abcam, ab86607) and VEGF (Abcam, ab46154) to visualize the protein status. Sections were stained with antibody against Ki67 (Abcam, ab28364) to test the level of cell proliferation. For CD31 staining, sections were permeabilized with 36 μg/ml proteinase K (Roche Diagnostics Corp.) and stained with antibody against CD31 (BD, Bioscience). Direct and indirect tyramide signal amplification kits (NEN Life Science Products, Inc.) were used to amplify staining signals. Sections were photographed at x200 magnification using an Olympus AX70 microscope. Vessel density (average of five fields) was determined with Image-Pro software.

### Statistical analysis

Differences between experimental groups were evaluated by one-way ANOVA and Tukey’s post-hoc test using SPSS11.5 software (SPSS, Chicago, IL). Statistical significance was based on a P-value of 0.05 (P<0.05, two-tailed test).

## SUPPLEMENTARY MATERIALS FIGURES


