# Supplementary material for: Oligomer procyanidins (F2) repress HIF-1α expression in human U87 glioma cells by inhibiting the EGFR/ AKT/mTOR and MAPK/ERK1/2 signaling pathways in vitro and in vivo
Source: Oncotarget. 2017 Jul 28;8(49):85252–62. doi: 10.18632/oncotarget.19654 (PMC5689607; doi:10.18632/oncotarget.19654)
Supplement: Supplementary file 1 [file oncotarget-08-85252-s001.pdf]

Oligomer procyanidins (F2) repress HIF-1α expression in human U87 glioma cells by inhibiting the EGFR/ AKT/mTOR and MAPK/ ERK1/2 signaling pathways *in vitro* and *in vivo*

SUPPLEMENTARY MATERIALS

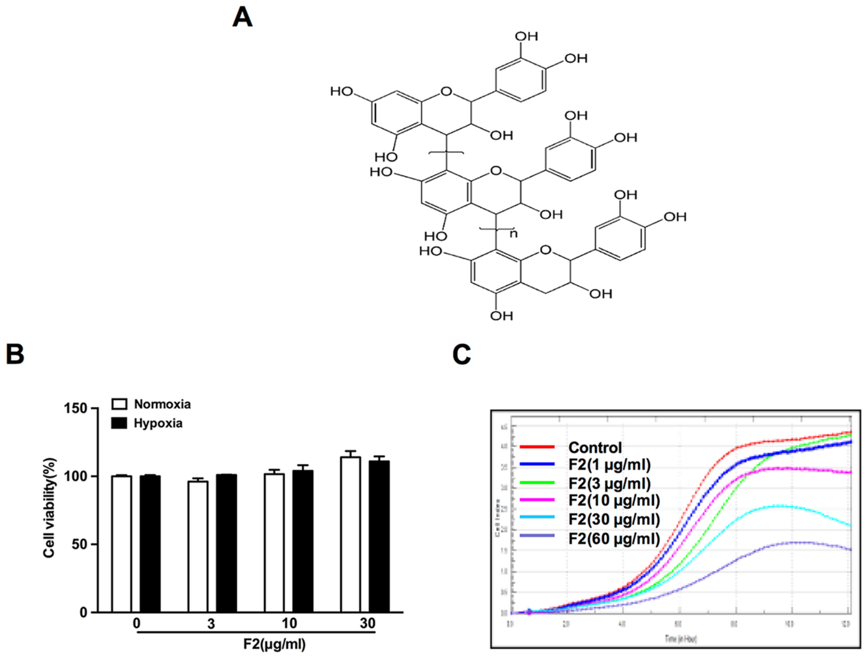

Supplementary Figure 1: The effect of F2 on cell viability and cell invasion. (A) General structure of oligomer procyanidins (F2). **(B)** Effects of F2 on the viability of U87 cells and HUVECs. Cells were treated with the indicated concentrations of F2 under normoxic and hypoxic conditions for 16 h. Cell viability was determined by SRB assay. Values are means ± SE of three experiments. **(C)** Transwell assays were used to evaluate the effect of F2 on the invasion ability of HUVECs.

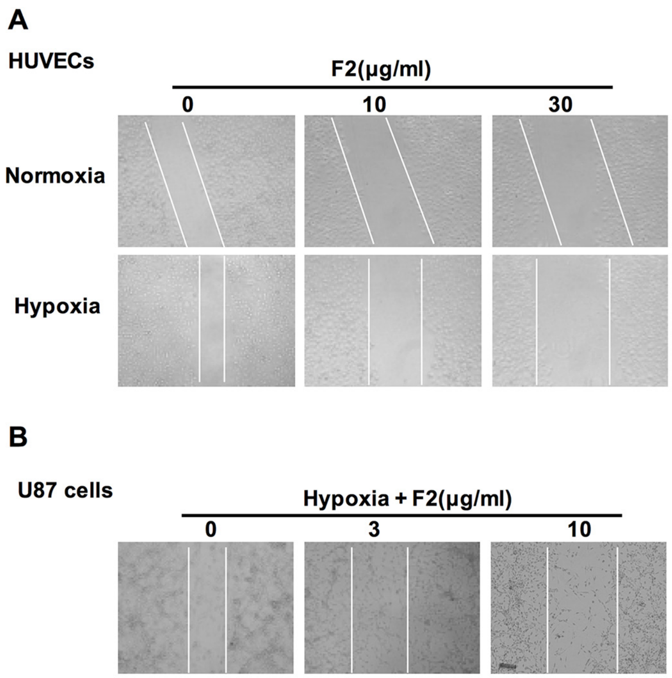

Supplementary Figure 2: The effect of F2 on the migration of HUVECs and U87 cells. **(A, B)** Wound healing assays were used to examine the effect of F2 on the migratory capacity of hypoxic HUVECs and U87 cells.
